# Multiplex qPCR for reliable detection and differentiation of *Burkholderia mallei* and *Burkholderia pseudomallei*

**DOI:** 10.1186/1471-2334-13-86

**Published:** 2013-02-14

**Authors:** Ingmar Janse, Raditijo A Hamidjaja, Amber CA Hendriks, Bart J van Rotterdam

**Affiliations:** 1 Laboratory for Zoonoses and Environmental Microbiology, National Institute for Public Health and the Environment (RIVM), Anthonie van Leeuwenhoeklaan 9, Bilthoven, MA, 3721, The Netherlands; 2 Diagnostic Laboratory for Infectious Diseases and Perinatal Screening, Centre for infectious Disease Control (CIB), National Institute for Public Health and the Environment (RIVM), Bilthoven, the Netherlands

**Keywords:** *Burkholderia mallei*, *Burkholderia pseudomallei*, Glanders, Melioidosis, Detection, qPCR, Sensitive detection, Internal amplification control

## Abstract

**Background:**

*Burkholderia mallei* and *B. pseudomallei* are two closely related species of highly virulent bacteria that can be difficult to detect. Pathogenic *Burkholderia* are endemic in many regions worldwide and cases of infection, sometimes brought by travelers from unsuspected regions, also occur elsewhere. Rapid, sensitive methods for identification of *B. mallei* and *B. pseudomallei* are urgently needed in the interests of patient treatment and epidemiological surveillance.

**Methods:**

Signature sequences for sensitive, specific detection of pathogenic *Burkholderia* based on published genomes were identified and a qPCR assay was designed and validated.

**Results:**

A single-reaction quadruplex qPCR assay for the detection of pathogenic *Burkholderia,* which includes a marker for internal control of DNA extraction and amplification, was developed. The assay permits differentiation of *B. mallei* and *B. pseudomallei* strains, and probit analysis showed a very low detection limit. Use of a multicopy signature sequence permits detection of less than 1 genome equivalent per reaction.

**Conclusions:**

The new assay permits rapid detection of pathogenic *Burkholderia* and combines enhanced sensitivity, species differentiation, and inclusion of an internal control for both DNA extraction and PCR amplification.

## Background

The ubiquitous Proteobacterial genus *Burkholderia* includes several animal and plant pathogens. Two closely related *Burkholderia* species cause severe, potentially fatal disease in humans. *Burkholderia mallei* is an obligate mammalian pathogen that causes glanders, a disease that is found in much of the world apart from North America, Europe and Australia. The disease mainly affects solipeds, but transmission to humans is possible through direct contact with animals and aerosols. Naturally infected human cases are reported only sporadically, but the causative agent is highly pathogenic under laboratory conditions and there have been several reports of laboratory-acquired infections [1]. *Burkholderia pseudomallei* is present in the environment and is a facultative pathogen that causes melioidosis, a glanders-like disease. It is a disease of humans and animals in all tropical and sub-tropical regions, but particularly South and Southeast Asia and northern Australia. Cases, included those brought by travelers, also occur outside endemic regions [2,3]. Glanders and melioidosis cause diagnostic problems in endemic regions, and even more so when imported into non-endemic areas due to a lack of awareness of these diseases there.

The variety of clinical manifestations means that the diagnosis of melioidosis or glanders cannot be based on symptomatic evidence alone and currently requires cultivation of the causative agent. This is a time-consuming process, and even more time is needed to confirm the species involved by means of biochemical tests. Moreover, misidentification due to the use of rapid biochemical methods has been reported [4,5].

Timely recognition of *B. mallei* and *B. pseudomallei* is vital for appropriate therapy, since both pathogens cause rapidly progressive diseases and are resistant to several antibiotics. These features, together with the relative ease with which these pathogens can be obtained and transmitted, the difficulties experienced in diagnosing the resultant diseases, and the fact that no effective protection through vaccination exists, have put them in the highest risk category of biothreat agents (classified as ‘Tier 1’ under the revised US select agents regulations, http://www.selectagents.gov). It is thus vital to have fast, sensitive methods for the identification of *B. mallei* and *B. pseudomallei*, both for patient treatment and for epidemiological surveillance and forensic investigation in the event of their deliberate release.

Several molecular tests using different detection platforms have been described for this purpose [6,7]. A real-time single-reaction assay for detection would however permit faster detection with less effort. Such assays for the detection and differentiation of *B. mallei* and *B. pseudomallei*, based on duplex hydrolysis probes for allelic discrimination, were recently described [6,8]. These assays did not include internal controls for DNA extraction and PCR amplification, however. Moreover, reliance on one signature sequence for detection of pathogenic *Burkholderia* may not be sufficiently specific, since *B. pseudomallei* and *B. mallei* display considerable genomic plasticity [9,10] and emerging novel strains will continue to challenge the coverage and sensitivity of these assays.

We have developed a single-reaction quadruplex qPCR assay for rapid, reliable detection of pathogenic *Burkholderia*. The assay combines enhanced sensitivity based on use of a specific multicopy sequence shared by both species, robust differentiation based on use of two different species-specific signature sequences, and enhanced reliability due to the incorporation of a marker that serves as internal control for DNA extraction and PCR amplification.

## Methods

### Design of primers and probes for multiplex hydrolysis probe assay

Both completed and unfinished genomes from *B. mallei* (10) and *B. pseudomallei* (29) available from public databases were analyzed by using the software package Kodon for management and analysis of sequences ( http://www.applied-maths.com) and the insignia web tool ( http://insignia.cbcb.umd.edu). Several potential signature sequences were identified for these organisms. The transposase ISBma2 was present in about 40–50 copies in *B. mallei* and about 5 copies in *B. pseudomallei*. Although this transposase has homologues in other organisms, a region of approximately 150 bp could be identified, which is present exclusively in *B. mallei* and *B. pseudomallei*, and not in *B. oklahomensis*. In addition, several unique signature sequences for differentiation of *B. mallei* and *B. pseudomallei* were identified. Out of these, the longest unique sequences were selected for primer and probe design. Both signature sequences corresponded to hypothetical proteins. The *B. pseudomalllei* signature sequence psu corresponded to locus BPSS1387 in the published genome of strain K96243 (Genebank accession number BX571966). This gene codes for a putative acetyltransferase, which is part of the type III secretion system-associated gene cluster. *B. mallei* signature sequence mau corresponded to locus BMA2524.1 in the published genome of strain ATCC 23344 (Genebank accession number CP000010). This gene codes for a phage integrase family protein.

The Cry1 gene of *Bacillus thuringiensis* was used as a signature sequences for the detection of this organism. Addition of these highly refractory spores to the assays served as internal control for DNA isolation and amplification (see also [11,12]). The software package Visual OMP ( http://www.DNAsoftware.com) was used to design a 4-target real-time PCR, as was described before [12]. An initial design yielded an unexpectedly high Cq for the multicopy sequence for two B. pseudomallei isolates (NCTC 4845 and NCTC 12939 T). Sequence analysis revealed a variation at one position of the probe annealing site, and a degeneracy was introduced to cover all strain variants (Table 1).

**Table 1:** Primers and probes for multiplex qPCR

***Organism***	***Target***	***Oligo function***	***Oligo name***	***Sequence 5'-3'***^***a***^
**B. pseudomallei + **	ISBma2	primer	Bumcpri_f	GCGGAAGCGGAAAAAGGG
B. mallei	ISBma2transposase	primer	Bumcpri_r	GCGGGTAGTCGAAGCTG
		probe	Tqpro_Bumc	**FAM**-TCRCCAGACGCAGCAGCAT-**BHQ1**
*B. pseudomallei*	Hypothetical	primer	psupri_f	GCGCGATCCGTCGAG
	protein	primer	psupri_r	AGCCGCTACGACGATTATG
		probe	Tqpro_psu	**JOE**-CCGCGACAATACGACCATCC-**BHQ1**
*B. mallei*	Hypothetical	primer	maupri3_f	GGCGAAAGAACGCGAAC
	protein	primer	maupri3_r	GCGTTCCACGATCAACTCT
		probe	Tqpro2_mau	**CF590**-CATCCCGCACCGTCCG-**BHQ2**
*B. thuringiensis*	Crystal protein	primer	Btpri_f	GCAACTATGAGTAGTGGGAGTAATTTAC
	gene	primer	Btpri_r	TTCATTGCCTGAATTGAAGACATGAG
		probe	Tqpro_Bt	**Cy5**-ACGTAAATACACT**-BHQ2-**TGATCCATTTGAAAAG**-P**

^a^ CFR590= CalFluor Red 590, BHQ= Black Hole quencher, P= phosporylation.

### PCR and real-time qPCR

Oligonucleotides were synthesized by Biolegio (Biolegio, Nijmegen, the Netherlands).

All qPCR reactions were carried out in a final volume of 20 µl containing iQ Multiplex Powermix (Bio-Rad, Veenendaal, the Netherlands), 200 nM of each primer and 100–300 nM hydrolysis probes and 3 µl of DNA template. Probe concentrations had been optimized to yield minimal spectral overlap between fluorescence level of the reporter dyes for each target in a multiplex assay and were 100, 200, 300 and 300 nM for FAM, JOE, CFR590 and Cy5 labeled probes respectively. The thermal cycling conditions were as follows: first enzyme activation at 95°C for 5 min, followed by amplification and detection by 45 thermocycles at 95°C for 5 sec and 60°C for 35 sec. Each real-time qPCR experiment included a negative (no template) control. Measurements were carried out on a LightCycler 480 (Roche, Almere, the Netherlands). Analyses were performed on the instruments software: LightCycler 480 Software release 1.5.0. SP3 and Cq values were calculated using the second derivative method. Color compensation was carried out according to the manufacturers’ guidelines.

### Bacterial isolates and genomic DNA preparation

The detection limits and specificities of the assays were evaluated using genomic materials from the bacterial strains and other sources displayed in Table 2. More details about the source and handling of the materials can be found in [12]. Lysates from the clinical isolates designated BD (Table 2) were prepared by boiling colonies cultivated on blood agar plates in water for 30 min. Autopsy materials were obtained from a melioidosis patient. A QIAamp DNA Mini kit (Qiagen, Crawley, UK) was used to extract DNA from liver, spleen, lung and prostate tissue samples. Spore suspensions of *B. thuringiensis* strain ATCC 29730 (var. galleriae Heimpel) that were used as internal controls, were obtained from Raven Biological Laboratories (Omaha, Nebraska, USA). These washed spores were counted by microscopy and then aliquotted and stored at 4°C. The amount of spores that needs to be added to samples to obtain suitable Cq values for this internal control must be determined empirically for each stock spore suspension. Ten-fold serial dilutions were made from the spore stock and DNA was extracted from 50 µl portions of each dilution by using the Nuclisens Magnetic Extraction Reagents (bioMérieux). The developed real-time qPCR assays were used to determine the amount of spores required for a Cq value between 32 and 35.

**Table 2:** Panel of organisms used for coverage and specificity analysis

**Species**	**Strain**	**Strain details**^**a**^	**Targets**^**b**^			
			**BuMC**	**psu**	**mau**	**cry1**
*Burkholderia*	NCTC 10229	Bird, 1961	14,5	-	19,2	-
*mallei*	NCTC 10230	Horse, 1961	14,6	-	19,3	-
	NCTC 10245	Horse, 1972	14,1	-	18,8	-
	NCTC 10247	Turkey, 1960	15,2	-	19,8	-
	NCTC 10248	Clinical isolate, Turkey, 1950	12,2	-	17,4	-
	NCTC 10260	Clinical isolate, Turkey, 1949	16,2	-	20,8	-
	NCTC 120	1920	14,8	-	19,4	-
	NCTC 3708	Mule, India, 1932	14,8	-	19,4	-
	NCTC 3709	Horse, India, 1932	15,0	-	19,3	-
	NCTC 12938 T	Clinical isolate	15,6	-	20,0	-
*Burkholderia*	NCTC 10274	Clinical isolate, Kuala Lumpur, 1962	16,3	18,5	-	-
*pseudomallei*	NCTC 10276	Clinical isolate, 1962	16,5	19,0	-	-
	NCTC 11642		14,7	18,2	-	-
	NCTC 1688	Rat, Malaysia, 1923	16,9	19,2	-	-
	NCTC 4845	infected laboratory monkey, Singapore,1935	-	18,8	-	-
	NCTC 4846	infected laboratory monkey, Singapore,1935	15,9	18,3	-	-
	NCTC 6700	Clinical isolate, 1942	15,9	18,7	-	-
	NCTC 7383	1948	16,0	18,7	-	-
	NCTC 7431	1948	16,0	18,3	-	-
	NCTC 8016	Sheep, Queensland, 1949	17,0	18,8	-	-
	NCTC 8707	Jordan, 1946	16,6	18,8	-	-
	NCTC 8708	Jordan, 1946	17,8	19,9	-	-
	NCTC 12939 T	Clinical isolate, USA, 1953	-	18,4	-	-
	BD08-00100	Clinical isolate, Netherlands, 2008^c^	17,0	18,3	-	-
	BD08-00103	Clinical isolate, Netherlands, 2008	18,8	20,2	-	-
	BD08-00268	Clinical isolate, Netherlands, 2008	16,2	19,0	-	-
	BD10-00211	Clinical isolate, Netherlands, 2010	-	18,6	-	-
	BD12-00016	Clinical isolate, Netherlands, 2012	18,5	21,1	-	-
	BD12-00217	Clinical isolate, Netherlands, 2012	19,6	22,2	-	-
*Burkholderia*	DSM 13276	Environmental sample, Thailand	-	-	-	-
*thailandensis*	CIP 106301	Soil, Thailand, 1994	-	-	-	-
	CIP 106302		-	-	-	-
*Bacillus anthracis*	NCTC 8234	Weybridge, 1951 (Sterne)	-	-	-	-
	NCTC 10340	Cow, Edinburgh, 1963 (Vollum)	-	-	-	-
*Francisella tularensis*	BD07-537	Clinical isolate, Netherlands, 2007	-	-	-	-
*subsp. holarctica (B)*						
*Yersinia pestis*	Kenya 164	Biovar antiqua, Kenya, <1952	-	-	-	-
	Harbin	Biovar mediaevalis, China, <1948	-	-	-	-
	Madagascar 34-94	Biovar orientalis, Madagascar	-	-	-	-
*Bacillus atrophaeus*	ATCC 9372		-	-	-	-
*Bacillus cereus*	ATCC 11778	NCIB Aberdeen, 1962	-	-	-	-
*Bacillus coagulans*		Purchased at Raven Labs, USA	-	-	-	-
*Bacillus megaterium*	ATCC 8245		-	-	-	-
*Bacillus pumilus*	ATCC 27142		-	-	-	-
*Bacillus subtilis*	ATCC 6633		-	-	-	-
*Bacillus thuringiensis*	ATCC 29730	var. galleriae Heimpel	-	-	-	20,4
*Enterobacter cloacae*	NCTC 13168		-	-	-	-
*Escherichia coli*	ATCC 25922	Clinical isolate, 1946	-	-	-	-
*Pseudomonas*	ATCC 15442		-	-	-	-
*aeruginosa*	ATCC 27853	Blood culture, 1969	-	-	-	-
*Klebsiella pneumoniae*	BD05-258	Clinical isolate, 2005	-	-	-	-
*Salmonella enterica*	ATCC 14028	Serovar Typhimurium.	-	-	-	-
*subsp. enterica*		liver, 1987	-	-	-	-
	ATCC 13076	Serovar Enteritidis	-	-	-	-
*Bos taurus*	0469	Netherlands, 2009	-	-	-	-
*Chrysops relictus*	I	Tissue, Netherlands, 2009	-	-	-	-
*Homo sapiens*	Volunteer 8	Blood, Netherlands, 2009	-	-	-	-
	Volunteer 10	Blood, Netherlands, 2009	-	-	-	-
*Equus ferus caballus*		Tissue, Netherlands, 2010	-	-	-	-
*Mus musculus*		Netherlands, 2009	-	-	-	-
*Ovis aries*	Twello 67	Slaughterhouse, Netherlands, 2009	-	-	-	-
*Rattus norvegicus*	08604	Netherlands, 2009	-	-	-	-
*Rattus rattus*	08402	Netherlands, 2009	-	-	-	-
*Sus scrofa*	566	Slaughterhouse, Netherlands, 2009	-	-	-	-

^a^The names of the countries or towns are those used at the time of the strain isolation and have been kept for strain designation.

^b^Numbers represent Cq values; - means no amplification. ^c^All clinical isolates from *B. pseudomallei* designated BD were isolated in the Netherlands from imported cases from Thailand.

### Limit of detection, efficiency, repeatability and internal control dynamic range

Characterization of qPCR performance was guided by the MIQE guidelines [13]. Validations were carried out using genomic DNA that was purified from culture lysates. Detection limits (LOD) for genomic DNA were determined by using purified DNA from cultures of *B.mallei* strain NCTC 10229 and *B. pseudomallei* strain NCTC 10276. The concentration of purified genomic DNA was measured by using the Quant-iT™ PicoGreen dsDNA detection kit (Invitrogen) and a Fluoroskan Ascent Microplate fluorometer (Thermo Scientific). Serial dilutions of genomic DNA were used to calculate LODs from the proportion of positive qPCRs at each dilution. Four replicates of 10 serial dilutions of genomic DNA were measured by qPCR. Based on the results, an additional measurement was performed on 4 replicates of 10 novel serial dilutions. The measurements included at least one dilution with all replicates positive and one with all replicates negative. A probit analysis was performed using SPSS Statistics 19.0.0 to calculate the DNA concentration that could be measured with 95% probability.

Efficiency and repeatability were calculated from the log-linear portion of the calibration curve, covering 6 orders of magnitude. Four replicate measurements were obtained from each dilution. Because the variation in Cqs at the lowest template concentration was relatively high, these values were excluded from the calculations.

To investigate the concentration range of internal control *B. thuringiensis* DNA that could be added to *Burkholderia* DNA without interfering with the detection of low pathogen concentrations, a dilution series of the internal control was made in the presence of a constant and low concentration of the pathogens. Genomic DNA from *Burkholderia mallei* or *B. pseudomallei* (14 and 48 fg/reaction, respectively) was mixed with serial dilutions from genomic DNA from *B. thuringiensis* (1.3∙10^1^ – 1.3?∙10^8^ fg/reaction). These DNA mixtures were amplified in triplicate by using the developed qPCR assays and the Cq values were plotted to investigate possible inhibition.

## Results

Three signature sequences were developed to permit sensitive detection and differentiation of *B. mallei* and *B. pseudomallei*. ISBma2 transposase is present in multiple copies in both species, thus enabling sensitive detection. Although homologs of this transposase occur in related organisms, a portion of this sequence was identified that is unique for *B. mallei* and *B. pseudomallei*. Two unique signature sequences (designated mau and psu respectively) differentiate between *B. mallei* and *B. pseudomallei* by their presence or absence. Table 1 shows the oligonucleotides designed for the detection of these signature sequences in the multiplex qPCR assay. This assay uses oligonucleotides to detect the *cry1* gene from *Bacillus thuringiensis*. Spores from this organism added before DNA extraction thus provide internal checks on the successful recovery and amplification of DNA. The specificity and strain coverage of the assay were validated with the aid of a panel of DNA from *B. mallei* and *B. pseudomallei* strains, a number of close relatives of these organisms, and several other bacterial and eukaryote strains (Table 2). The assay was able to discriminate correctly between the two species. Clinical samples were obtained from the melioidosis patient from whom *B. pseudomallei* strain BD10-00211 had been isolated. Samples derived from the liver and spleen tested positive only for signature sequence psu, at Cqs of 37.8 and 34.9 respectively. The enhanced sensitivity of detection of pathogenic *Burkholderia* with the aid of the multicopy target (BuMC) was demonstrated by the Cq values for this target, which were clearly lower than those for the single-copy targets (a difference of 4.4 – 5.2 for *B. mallei* and 1.3 – 3.5 for *B. pseudomallei*). The multicopy sequence could not be detected in three of the *B. pseudomallei* strains (NCTC 4845, NCTC 12939 T and BD10-00211).

All standard curves had an R^2^ of >0.998 and showed high amplification efficiencies and linearity for the different targets, over at least 6 orders of magnitude (Table 3). Calculation of the limit of detection (LOD) using probit analysis showed high sensitivities of 0.2 and 3.7 fg for *B. mallei* and *B. pseudomallei* respectively (based on use of the multicopy target BuMC). 

**Table 3:** Assay performance

***Organism***^***a***^	***Target***	***Efficiency (%)***	***Linear range (fg/reaction)***	***Repeatability (SD of C***_***q***_***)***^***b***^	***LOD gDNA (fg/reaction)***^**c**^
*B. mallei*	BuMC	99,0	1.4·10^-1^ – 1.4·10^6^	0,072	0.2
	mau	98,7	1.4·10^0^ – 1.4·10^6^	0,080	4.5
*B. pseudomallei*	BuMC	96,6	1·10^0^ - 1·10^7^	0,085	3,7
	psu	99,2	1·10^1^ – 1·10^7^	0,077	57

^a^ Organism from which genomic DNA was used for assessment of assay performance.

^b^ Values represent the average from the standard deviations calculated at 6 different dilutions from 4 replicate Cq measurements.

^c^ Values displayed represent the lowest DNA concentration at which 95% of the positive samples are detected, as calculated by using probit analysis.

**Figure 1:**
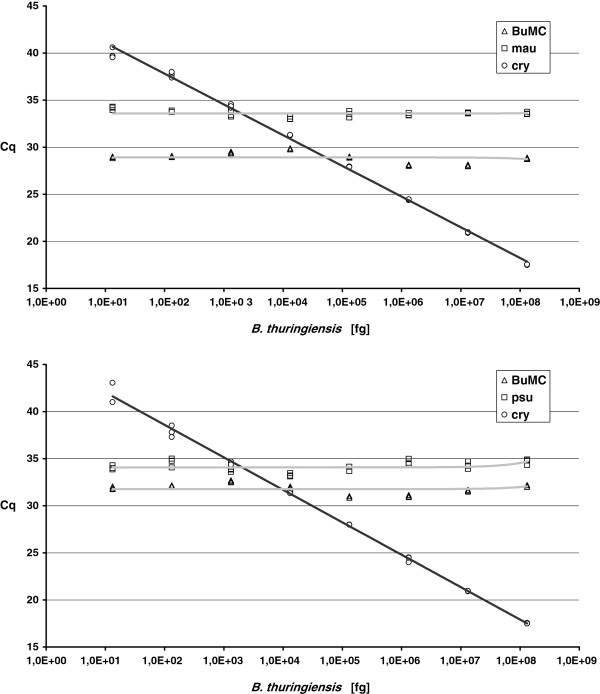
**Effect of increasing amounts of internal control on qPCR detection.** A concentration range of genomic DNA from *B. thuringiensis* was mixed with a constant and low concentration of 14 fg *B. mallei* gDNA (A) or 48 *B. pseudomallei* DNA (B), and measured by using the developed multiplex qPCR.

To confirm that the addition of the internal control has no effect on pathogen detection, a dilution series of *B. thuringiensis* DNA was made in the presence of a constant and very low concentration of *Burkholderia* DNA. As shown in Figure 1, the detection of all signature sequences of *B. mallei* and *B. pseudomallei* was unaffected by the presence of the internal control DNA, even when the latter was present at an excess of 10^5^:1. 

## Discussion

Methods for the rapid, specific identification of pathogenic *Burkholderia* species are important for timely recognition of glanders or melioidosis in patients with general clinical symptoms that could fit various diseases. Both *B. pseudomallei* and *B. mallei* are intrinsically resistant to many widely-used antibiotics, and susceptibilities differ between the two species [6,14]. Identification of and differentiation between the two pathogens could thus help healthcare workers to choose the right antibiotics to treat infected patients.

In regions where these species are endemic, infection with *Burkholderia mallei* or *B. pseudomallei* can often be pinpointed by consideration of prevalences and infection risk factors. The presence of the pathogens is less evident in non-endemic areas. Numerous cases of imported melioidosis have been reported [3,6], and are likely to continue to occur as travel and trade increase. Fast diagnostic methods are therefore essential for use in non-endemic areas, not only to support appropriate patient treatment but also to ensure the safety of laboratory workers culturing unknown organisms for diagnostic purposes. Another scenario in which rapid identification of pathogenic *Burkholderia* including species distinction is a challenging but essential task is that of deliberate release of these biothreat agents, where appropriate assay methods may be needed e.g. for forensic tracking.

The assay presented in this paper permits sensitive, reliable detection of pathogenic *Burkholderia*, thanks to the use of qPCR (real-time polymerase chain reaction). It reduces false-negative measurements, thanks to the inclusion of an internal control and the high sensitivity that was achieved.

The idea of adding spores from *B. thuringiensis* as internal controls was based on their properties as highly refractory biological structures. The detection of the *B. thuringiensis* signature sequence cry1 guarantees successful DNA extraction and amplification from any microorganism in the sample [11,12]. The target BuMC was used as a sensitive, specific indicator for the presence of pathogenic *Burkholderia* strains. The selection of this target based on its presence in multiple copies in all 21 publicly available genomes and its usefulness for sensitive detection was evidenced by the in vitro validation showing lower Cq values (Table 2). However, the benefit of more sensitive detection did not hold for all strains, since BuMC could not be amplified from three of the *B. pseudomallei* strains tested (Table 2). The absence of this target from the clinical samples is congruent with its absence from strain BD10-00211, which had been isolated from the patient from which the clinical samples were taken. It is possible that the rather diverse species *B. pseudomallei*[9] contains a phylogenetic cluster that has the absence of the targeted ISBma2 transposase gene as a common feature. Further research is required to substantiate this assumption, however.

We did not find any strains that were not detected by the species-specific signature sequences we developed. However, probe design is always limited by the available sequences and strains. Hence, the possibility that some strain exists or could arise which does not possess one of the signature sequences used in this assay cannot be excluded. This is true of any assay, however, and the multiplex qPCR assay described here has the advantage of possessing two signatures for each strain, thus reducing the risk that some strains will escape detection.

The measured linearity and efficiency show that the qPCR assay is very suitable for quantitative measurement. The calculated LODs were very low, particularly when based on multicopy target sequence BuMC. The detection limits of 0.2 fg per reaction for *B. mallei* and 3.7 fg per reaction for *B. pseudomallei* (Table 3) correspond to approximately 0.03 and 0.5 genomic equivalents respectively. The LODs were lower than or similar to those reported for other assays [6,8,14-19]. However, it is difficult to make a direct comparison between reported LODs due to the differences between the methods used to measure and calculate them, and to measure the DNA concentration of the standards. We used the DNA intercalating dye picogreen for accurate determination of the concentration of double-stranded DNA in our stocks and probit analysis as a basis for robust calculation of the concentration at which the probability of detecting the target is 95% [11].

The high reliability and sensitivity of the qPCR assay described here make it very useful for screening of samples containing few organisms and potential inhibitors, as is the case in many environmental and clinical samples. It can furthermore be used to supplement other assays, including molecular assays based on other signature sequences, for definitive identification. *Burkholderiaceae* are highly recombining organisms [6,9,10,18] and emerging novel strains will continue to challenge the coverage and sensitivity of detection assays. This qPCR assay offers the potential of continuing to meet this challenge effectively in the foreseeable future.

## Conclusions

We designed an assay for rapid and reliable detection of pathogenic *Burkholderia spp*. The qPCR assay reduces false-negative measurements, due to the inclusion of an internal control and to the high sensitivity that was achieved. Based on a multicopy signature sequence, detection of less than 1 genome equivalent per reaction was possible. Species could be differentiated based on two species-specific signature sequences. The multiplex format limits sample handling, labor and cost per sample. This makes the assay very useful for reliable screening of environmental and clinical samples.

## Competing interests

The authors declare that they have no competing interests.

## Authors’ contributions

IJ conceived of and designed the study, performed bioinformatics and probe design, analyzed the data and drafted the manuscript. RAH performed the experimental work and participated in study design and data analysis. AH performed experimental work. BJvR coordinated the work. All authors read and approved the final manuscript.

## Pre-publication history

The pre-publication history for this paper can be accessed here:

http://www.biomedcentral.com/1471-2334/13/86/prepub
